# 4-(*o*-Tolyl­amino)­benzaldehyde

**DOI:** 10.1107/S1600536810040626

**Published:** 2010-10-20

**Authors:** Li-Ying Wang, Yong-Sheng Xie, Ren-Min Wu, Hua Zuo

**Affiliations:** a College of Pharmaceutical Sciences, Southwest University, Chongqing 400715, People’s Republic of China; b School of Chemical and Environmental Engineering, Chongqing Three Gorges University, Chongqing 404100, People’s Republic of China

## Abstract

In the title compound, C_14_H_13_NO, the dihedral angle between the aromatic rings is 49.64 (18)°. The crystal structure is stabilized by N—H⋯O, C—H⋯O and C—H⋯π hydrogen bonds.

## Related literature

For applications and bioactivity of diaryl­amines, see: Ohta *et al.* (2008 ▶); Li *et al.* (2008 ▶).
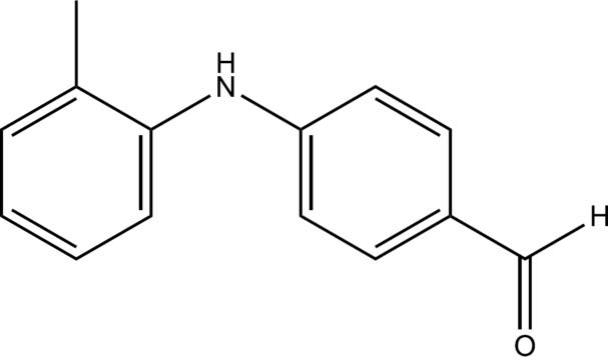


## Experimental

### 

#### Crystal data

C_14_H_13_NO
*M* *_r_* = 211.25
Orthorhombic, 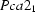

*a* = 14.193 (10) Å
*b* = 10.699 (10) Å
*c* = 7.677 (6) Å
*V* = 1165.9 (16) Å^3^
*Z* = 4
Mo *K*α radiation
μ = 0.08 mm^−1^
*T* = 273 K
0.20 × 0.15 × 0.05 mm

#### Data collection

Bruker SMART CCD area-detector diffractometer
6127 measured reflections
1397 independent reflections
1140 reflections with *I* > 2σ(*I*)
*R*_int_ = 0.033

#### Refinement

*R*[*F* ^2^ > 2σ(*F* ^2^)] = 0.050
*wR*(*F* ^2^) = 0.157
*S* = 1.06
1397 reflections
153 parameters
1 restraint
H atoms treated by a mixture of independent and constrained refinement
Δρ_max_ = 0.22 e Å^−3^
Δρ_min_ = −0.21 e Å^−3^

### 

Data collection: *SMART* (Bruker, 2004 ▶); cell refinement: *SAINT* (Bruker, 2004 ▶); data reduction: *SAINT*; program(s) used to solve structure: *SHELXS97* (Sheldrick, 2008 ▶); program(s) used to refine structure: *SHELXL97* (Sheldrick, 2008 ▶); molecular graphics: *SHELXTL* (Sheldrick, 2008 ▶); software used to prepare material for publication: *SHELXL97*.

## Supplementary Material

Crystal structure: contains datablocks I, global. DOI: 10.1107/S1600536810040626/si2297sup1.cif

Structure factors: contains datablocks I. DOI: 10.1107/S1600536810040626/si2297Isup2.hkl

Additional supplementary materials:  crystallographic information; 3D view; checkCIF report

## Figures and Tables

**Table 1:** Hydrogen-bond geometry (Å, °) *Cg*1 is the centroid of the C8–C13 tolyl ring.

*D*—H⋯*A*	*D*—H	H⋯*A*	*D*⋯*A*	*D*—H⋯*A*
N1—H1⋯O1^i^	0.79 (4)	2.32 (4)	3.099 (5)	171 (3)
C14—H14*A*⋯O1^i^	0.96	2.48	3.334 (6)	148
C9—H9⋯*Cg*1^ii^	0.93	2.95	3.603 (5)	128

Symmetry codes: (i) 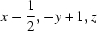; (ii) 
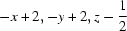
.

**Fig. 1.**
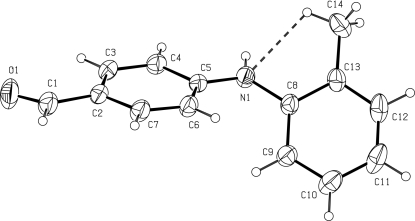
The molecular structure of the title compound, with displacement ellipsoids drawn at the 30% probability level.

**Fig. 2.**
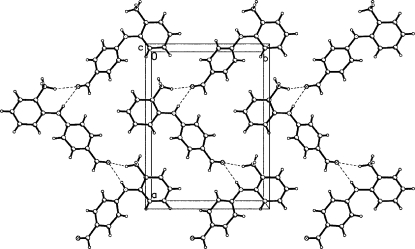
A section of the crystal packing of the title compound. Intermolecular hydrogen bonds are shown by dashed lines.

## supplementary crystallographic information

### Comment

Diarylamines represent an important class of compounds due to their wide applications and special pharmacological activities (Ohta *et al.* 2008; Li *et al.* (2008).). We report here the synthesis and the crystal structure of the title compound, C_14_H_13_NO, which consists of benzaldehyde and tolyl groups attached at the terminal nitrogen atoms (Fig. 1). The dihedral angle between the aromatic rings is 49.64 (18)°. The N1, C14 and H14A atoms are coplanar with the phenyl ring C8 to C13, with deviations of -0.053 (3) Å, -0.076 (4) Å, and -0.07 (1) Å from the ring plane, respectively. The non-planar conformation of the title molecule is not only due to the intramolecular C14-H14A···N1 hydrogen bond, but also owing to the repulsion of H14A and H1 together with packing effects and intermolecular interactions (Fig. 1 and Table 1).In the crystal, zigzag chains are formed along *a* through the intermolecular N—H···O and C—H···O hydrogen bonds (Fig. 2 and Table 1). The molecules are also stabilized by weak C—H···π interactions (Table 1: Cg1^ii^ is the centroid of the tolyl ring C8 - C13).

### Experimental

To a magnetically stirred solution of *o*-toluidine (1.0 mmol) and Cs_2_CO_3_ (3.2 mmol) in dry DMF cooled by ice bath were added chloroacetyl chloride (1.2 mmol) and 4-hydroxybenzaldehyde (1.0 mmol). The reaction mixture was then stirred for 30 min at room temperature and placed into a microwave oven (600 W, 423K) and irradiated for 35 min. The solvent was removed under vacuum and water (20 ml) was added into the residue. The mixture was then extracted by ethyl acetate (4 *x* 30 ml). The combined organic layers were dried over anhydrous MgSO_4_ and evaporated under vacuum to give the crude product, which was purified by column chromatography on silica gel using ethyl acetate/petroleum ether (yield 89%). Crystals suitable for X-ray diffraction were obtained by slow evaporation of a solution of the solid dissolved in ethyl acetate/petroleum ether at room temperature for 4 days.

### Refinement

All H atoms were positioned geometrically and refined using a riding model, with C—H = 0.93 Å for aryl and 0.96 Å for methyl H atoms, and with *U*_iso_(H) =1.2*U*_eq_(C) for aryl H atoms, and 1.5*U*_eq_(C) for methyl H atoms. The C1- and N1-bound H-atoms were located in a difference Fourier map, the *U*_iso_ values were freely refined. In the absence of significant anomalous dispersion effects, Friedel pairs were averaged.

### Crystal data

**Table:**

C_14_H_13_NO	*D*_x_ = 1.204 Mg m^−^^3^
*M**_r_* = 211.25	Mo *K*α radiation, λ = 0.71073 Å
Orthorhombic, *P**c**a*2_1_	Cell parameters from 2572 reflections
*a* = 14.193 (10) Å	θ = 2.4–26.6°
*b* = 10.699 (10) Å	µ = 0.08 mm^−^^1^
*c* = 7.677 (6) Å	*T* = 273 K
*V* = 1165.9 (16) Å^3^	Plate, brown
*Z* = 4	0.20 × 0.15 × 0.05 mm
*F*(000) = 448	

### Data collection

**Table:**

Bruker SMART CCD area-detector diffractometer	1140 reflections with *I* > 2σ(*I*)
Radiation source: fine-focus sealed tube	*R*_int_ = 0.033
graphite	θ_max_ = 27.4°, θ_min_ = 1.9°
phi and ω scans	*h* = −18→14
6127 measured reflections	*k* = −13→13
1397 independent reflections	*l* = −6→9

### Refinement

**Table:**

Refinement on *F*^2^	Primary atom site location: structure-invariant direct methods
Least-squares matrix: full	Secondary atom site location: difference Fourier map
*R*[*F*^2^ > 2σ(*F*^2^)] = 0.050	Hydrogen site location: inferred from neighbouring sites
*wR*(*F*^2^) = 0.157	H atoms treated by a mixture of independent and constrained refinement
*S* = 1.06	*w* = 1/[σ^2^(*F*_o_^2^) + (0.1027*P*)^2^ + 0.0736*P*] where *P* = (*F*_o_^2^ + 2*F*_c_^2^)/3
1397 reflections	(Δ/σ)_max_ < 0.001
153 parameters	Δρ_max_ = 0.22 e Å^−^^3^
1 restraint	Δρ_min_ = −0.21 e Å^−^^3^

### Special details

**Table:**

Geometry. All e.s.d.'s (except the e.s.d. in the dihedral angle between two l.s. planes) are estimated using the full covariance matrix. The cell e.s.d.'s are taken into account individually in the estimation of e.s.d.'s in distances, angles and torsion angles; correlations between e.s.d.'s in cell parameters are only used when they are defined by crystal symmetry. An approximate (isotropic) treatment of cell e.s.d.'s is used for estimating e.s.d.'s involving l.s. planes.
Refinement. Refinement of *F*^2^ against ALL reflections. The weighted *R*-factor *wR* and goodness of fit *S* are based on *F*^2^, conventional *R*-factors *R* are based on *F*, with *F* set to zero for negative *F*^2^. The threshold expression of *F*^2^ > σ(*F*^2^) is used only for calculating *R*-factors(gt) *etc*. and is not relevant to the choice of reflections for refinement. *R*-factors based on *F*^2^ are statistically about twice as large as those based on *F*, and *R*- factors based on ALL data will be even larger.

### Fractional atomic coordinates and isotropic or equivalent isotropic displacement parameters (Å^2^)

**Table:**

	*x*	*y*	*z*	*U*_iso_*/*U*_eq_	
C6	1.07328 (19)	0.7362 (2)	0.3779 (5)	0.0546 (7)	
H6	1.0775	0.8092	0.4432	0.066*	
C5	0.99125 (16)	0.7114 (2)	0.2798 (4)	0.0476 (6)	
N1	0.91447 (16)	0.7907 (2)	0.2760 (4)	0.0567 (7)	
C8	0.91320 (18)	0.9212 (2)	0.3158 (4)	0.0496 (7)	
C4	0.98692 (19)	0.5966 (2)	0.1871 (5)	0.0534 (7)	
H4	0.9324	0.5758	0.1265	0.064*	
C3	1.0628 (2)	0.5154 (2)	0.1858 (5)	0.0548 (7)	
H3	1.0591	0.4418	0.1217	0.066*	
C2	1.14506 (19)	0.5424 (2)	0.2797 (5)	0.0531 (7)	
C13	0.83034 (19)	0.9730 (2)	0.3886 (5)	0.0557 (7)	
O1	1.23816 (19)	0.36802 (18)	0.1876 (6)	0.0897 (10)	
C1	1.2296 (2)	0.4612 (3)	0.2747 (6)	0.0660 (9)	
C7	1.1475 (2)	0.6520 (2)	0.3770 (5)	0.0581 (8)	
H7	1.2006	0.6694	0.4437	0.070*	
C9	0.9897 (2)	0.9981 (3)	0.2718 (5)	0.0577 (7)	
H9	1.0431	0.9638	0.2204	0.069*	
C14	0.7450 (3)	0.8922 (3)	0.4262 (8)	0.0821 (11)	
H14A	0.7589	0.8069	0.3968	0.123*	
H14B	0.6926	0.9208	0.3580	0.123*	
H14C	0.7295	0.8976	0.5477	0.123*	
C12	0.8298 (3)	1.1013 (3)	0.4195 (6)	0.0742 (10)	
H12	0.7765	1.1371	0.4693	0.089*	
C10	0.9854 (2)	1.1256 (3)	0.3053 (7)	0.0728 (11)	
H10	1.0365	1.1764	0.2781	0.087*	
C11	0.9052 (3)	1.1773 (3)	0.3790 (7)	0.0814 (12)	
H11	0.9023	1.2627	0.4011	0.098*	
H1A	1.286 (2)	0.483 (3)	0.339 (5)	0.063 (9)*	
H1	0.869 (3)	0.755 (3)	0.244 (5)	0.070 (11)*	

### Atomic displacement parameters (Å^2^)

**Table:**

	*U*^11^	*U*^22^	*U*^33^	*U*^12^	*U*^13^	*U*^23^
C6	0.0502 (14)	0.0407 (13)	0.0730 (19)	0.0008 (10)	−0.0053 (14)	−0.0097 (14)
C5	0.0395 (11)	0.0388 (12)	0.0645 (17)	−0.0005 (9)	0.0042 (12)	−0.0001 (14)
N1	0.0375 (10)	0.0445 (11)	0.0881 (19)	−0.0002 (9)	−0.0052 (12)	−0.0093 (14)
C8	0.0417 (12)	0.0427 (13)	0.0645 (18)	0.0050 (10)	−0.0062 (12)	−0.0031 (13)
C4	0.0441 (12)	0.0446 (12)	0.0716 (19)	−0.0062 (10)	−0.0046 (14)	−0.0073 (15)
C3	0.0586 (15)	0.0355 (11)	0.0704 (18)	−0.0036 (11)	0.0043 (15)	−0.0069 (15)
C2	0.0487 (13)	0.0375 (11)	0.0730 (19)	0.0034 (10)	0.0040 (14)	0.0039 (14)
C13	0.0469 (14)	0.0554 (15)	0.0646 (18)	0.0144 (11)	−0.0045 (14)	0.0001 (15)
O1	0.0794 (16)	0.0617 (12)	0.128 (3)	0.0276 (12)	−0.0038 (18)	−0.0164 (18)
C1	0.0559 (17)	0.0496 (15)	0.093 (3)	0.0101 (13)	−0.0029 (18)	0.0025 (19)
C7	0.0478 (14)	0.0446 (13)	0.082 (2)	0.0010 (11)	−0.0105 (15)	−0.0024 (15)
C9	0.0470 (13)	0.0486 (13)	0.077 (2)	0.0005 (10)	−0.0054 (15)	0.0012 (16)
C14	0.0541 (16)	0.090 (2)	0.102 (3)	0.0158 (18)	0.0239 (18)	0.008 (2)
C12	0.0644 (19)	0.0643 (18)	0.094 (3)	0.0275 (15)	−0.0133 (19)	−0.015 (2)
C10	0.0631 (17)	0.0484 (15)	0.107 (3)	−0.0040 (13)	−0.022 (2)	0.006 (2)
C11	0.083 (2)	0.0442 (15)	0.117 (3)	0.0170 (17)	−0.035 (2)	−0.010 (2)

### Geometric parameters (Å, °)

**Table:**

C6—C7	1.386 (4)	C13—C12	1.393 (4)
C6—C5	1.412 (4)	C13—C14	1.516 (5)
C6—H6	0.9300	O1—C1	1.207 (5)
C5—N1	1.381 (3)	C1—H1A	0.97 (4)
C5—C4	1.421 (4)	C7—H7	0.9300
N1—C8	1.429 (4)	C9—C10	1.390 (5)
N1—H1	0.80 (4)	C9—H9	0.9300
C8—C9	1.403 (4)	C14—H14A	0.9600
C8—C13	1.415 (4)	C14—H14B	0.9600
C4—C3	1.383 (4)	C14—H14C	0.9600
C4—H4	0.9300	C12—C11	1.381 (6)
C3—C2	1.403 (4)	C12—H12	0.9300
C3—H3	0.9300	C10—C11	1.386 (6)
C2—C7	1.391 (4)	C10—H10	0.9300
C2—C1	1.481 (4)	C11—H11	0.9300
			
C7—C6—C5	120.1 (2)	O1—C1—C2	125.5 (4)
C7—C6—H6	119.9	O1—C1—H1A	113.4 (19)
C5—C6—H6	119.9	C2—C1—H1A	121.0 (19)
N1—C5—C6	123.1 (2)	C6—C7—C2	122.2 (3)
N1—C5—C4	119.1 (2)	C6—C7—H7	118.9
C6—C5—C4	117.7 (2)	C2—C7—H7	118.9
C5—N1—C8	127.2 (2)	C10—C9—C8	119.8 (3)
C5—N1—H1	111 (2)	C10—C9—H9	120.1
C8—N1—H1	122 (2)	C8—C9—H9	120.1
C9—C8—C13	120.6 (2)	C13—C14—H14A	109.5
C9—C8—N1	120.7 (2)	C13—C14—H14B	109.5
C13—C8—N1	118.5 (2)	H14A—C14—H14B	109.5
C3—C4—C5	120.8 (3)	C13—C14—H14C	109.5
C3—C4—H4	119.6	H14A—C14—H14C	109.5
C5—C4—H4	119.6	H14B—C14—H14C	109.5
C4—C3—C2	121.0 (3)	C11—C12—C13	122.5 (3)
C4—C3—H3	119.5	C11—C12—H12	118.7
C2—C3—H3	119.5	C13—C12—H12	118.7
C7—C2—C3	118.0 (2)	C11—C10—C9	120.2 (3)
C7—C2—C1	119.3 (3)	C11—C10—H10	119.9
C3—C2—C1	122.7 (3)	C9—C10—H10	119.9
C12—C13—C8	117.3 (3)	C12—C11—C10	119.6 (3)
C12—C13—C14	121.7 (3)	C12—C11—H11	120.2
C8—C13—C14	121.1 (2)	C10—C11—H11	120.2

### Hydrogen-bond geometry (Å, °)

**Table:**

Cg1 is the centroid of the C8–C13 tolyl ring.

**Table:**

*D*—H···*A*	*D*—H	H···*A*	*D*···*A*	*D*—H···*A*
C14—H14A···N1	0.96	2.40	2.880 (6)	110
N1—H1···O1^i^	0.79 (4)	2.32 (4)	3.099 (5)	171 (3)
C14—H14A···O1^i^	0.96	2.48	3.334 (6)	148
C9—H9···Cg1^ii^	0.93	2.95	3.603 (5)	128

Symmetry codes:  (i) *x*−1/2, −*y*+1, *z*; (ii) −*x*+2, −*y*+2, *z*−1/2.
